# Associations between changes in caregiver’s and child’s weight status in a community-based obesity intervention programme

**DOI:** 10.1038/s41366-022-01121-3

**Published:** 2022-04-29

**Authors:** Thomas S. Hadley, Tami L. Cave, José G. B. Derraik, Paul L. Hofman, Yvonne C. Anderson

**Affiliations:** 1 Department of Paediatrics, Taranaki District Health Board, New Plymouth, New Zealand; 2 Liggins Institute, University of Auckland, Auckland, New Zealand; 3 Department of Paediatrics: Child and Youth Health, Faculty of Medical and Health Sciences, University of Auckland, Auckland, New Zealand; 4 Starship Children’s Health, Auckland District Health Board, Auckland, New Zealand; 5 enAble Institute, Faculty of Health Sciences, Curtin University, Bentley, WA Australia; 6 Telethon Kids Institute, Perth Children’s Hospital, Nedlands, WA Australia; 7 Community Health, Child and Adolescent Health Service, Perth, WA Australia

**Keywords:** Obesity, Translational research

## Abstract

**Objectives:**

We examined whether caregivers of children/adolescents enroled in a randomised controlled trial (RCT) of a family-centred intervention indirectly achieved reductions in body mass index (BMI), and if these were associated with changes in their children’s BMI.

**Methods:**

RCT participants were New Zealand children/adolescents aged 4.8–16.8 years with BMI ≥ 98th percentile or >91st with weight-related comorbidities. Participants and accompanying caregivers were assessed at baseline, 12, and 24 months.

**Results:**

Overall, caregivers’ BMI was unchanged at 12 or 24 months. Among Māori participants, reductions in caregivers’ BMI at 12 months were associated with reductions in their children’s BMI SDS at 12 (*r* = 0.30; *p* = 0.038) and 24 months (*r* = 0.39; *p* = 0.009). Further, children identifying as Māori whose caregivers’ BMI decreased at 12 months had greater BMI SDS reductions at 12 months [﻿−0.30 (95% CI −0.49, −0.10); *p* = 0.004] and 24 months [﻿−0.39 (95% CI −0.61, −0.16); *p* = 0.001] than children of caregivers with increased/unchanged BMI.

**Conclusions:**

This intervention programme for children/adolescents with obesity did not indirectly reduce caregiver weight status. However, reductions in caregivers’ BMI were key to BMI SDS reductions among Māori participants. Given the intergenerational nature of obesity, our findings highlight the importance of culturally relevant, family-focused programmes to achieve clinically meaningful improvements in weight status across the family.

## Introduction

In recent decades, there has been a marked rise in the prevalence of childhood overweight and obesity worldwide, which increased nearly fivefold from ≈4% in 1974 to just over 18% in 2016 [1]. Aotearoa/New Zealand (henceforth referred to as New Zealand) is ranked 2nd in the Organisation for Economic Cooperation and Development (OECD) for obesity prevalence [2]. Of particular concern is the over-representation of children who identify as Māori, Pacific, and those from most deprived households in obesity statistics compared to their counterparts [3]. In response to this global issue, the World Health Organization published a report in 2016 with recommendations to end childhood obesity [4]. It recommended that family-focused behavioural lifestyle interventions be provided to address obesity in children and young people [4].

Whānau Pakari is a multi-disciplinary, family-centred assessment and intervention programme in Taranaki (New Zealand), with a randomised controlled trial (RCT) initially embedded within the programme [5]. Two hundred and three children aged 5–16 years were randomised to a high-intensity intervention group (6-monthly home-based assessments with weekly group sessions for 12 months) or a minimal-intensity group (6-monthly home-based assessments with advice) [6]. In the RCT, there was an observed reduction in body mass index (BMI) standard deviation score (SDS) at 12 months, irrespective of intervention intensity [6]. High attendance in the high-intensity intervention resulted in sustained treatment effects with improvements in multiple health measures at 24 months, even though the overall BMI SDS reduction was lost [7]. Past research on family-based interventions reported a decrease in the BMI of accompanying adults [8]. Parental BMI has also been shown to predict the rate of change in their children’s BMI [9]. However, compared to Whānau Pakari, these studies tended to have families with a lower adult BMI at baseline and programmes with shorter duration and shorter follow-up [8, 9]. Therefore, in these secondary analyses, we examined whether caregivers of children in the Whānau Pakari programme indirectly achieved reductions in BMI over time. Further, we assessed whether changes in the caregivers’ BMI were associated with changes in the participants’ BMI SDS.

## Methods

Ethics approval for the RCT was provided by the Central Health and Disability Ethics Committee (CEN/11/09/054). Written and verbal consents/assents were obtained from all participants or their guardians. Trial registration was with the Australian New Zealand Clinical Trials Registry (ANZCTR: 12611000862943).

The detailed design of the Whānau Pakari RCT has been described previously [5, 6]. Briefly, it was an unblinded RCT in Taranaki, a region with a population of 23,139 children aged 0–15 years in 2013, of whom 28% identified as Māori (New Zealand’s indigenous peoples) [10]. Participants were recruited in 2012–2014, were aged 4.8–16.8 years, and had BMI ≥ 98th percentile or >91st percentile with weight-related comorbidities.

Assessments were undertaken in the home at baseline, 12 months, and 24 months. Where consent was given, data were recorded for the caregiver present at each assessment. Here, caregiver is defined as the adult accompanying the participant to assessments, who was primarily involved in the child/adolescent’s care. Only data for the same accompanying adult recorded at each assessment were included in this study. At baseline, demographic data were recorded, including the participant’s age, sex, and ethnicity, with household deprivation levels estimated using the NZ Index of Deprivation 2006 (NZDep2006) [11].

Height was measured using a Seca 213 portable stadiometer (SECA, Hamburg, Germany) to the nearest mm. Weight was measured using Seca 813 digital scales to 0.1 kg, with BMI subsequently calculated. For children and adolescents, BMI SDS was derived using the KIGS auxology software (Pfizer Endocrine Care™) based on UK Cole normative data [12].

### Statistical analyses

Linear associations between the accompanying adult’s BMI and their child’s BMI SDS at baseline were examined with Pearson’s correlation coefficients and simple linear regressions. A linear mixed model was subsequently run, adjusting for child’s sex, age, household deprivation (NZDep2006 in quintiles), and ethnicity (Māori/Non-Māori). Family ID was also included as a random factor to account for the non-independence of siblings. Possible changes in caregivers’ BMI at 12 and 24 months were assessed using paired t-tests.

The study population was then stratified into two groups according to caregiver’s BMI change (ΔcBMI) at 12 months (reduced vs unchanged or increased BMI). Linear mixed models were run with the child’s change in BMI SDS (ΔBMI SDS) at 12 or 24 months as the outcome. Models included ΔcBMI group, ethnicity, an interaction term (ΔcBMI group*ethnicity), sex, household deprivation, the child’s BMI SDS and age at baseline, and family ID (random factor). The ΔcBMI group*ethnicity interaction term was statistically significant in both models, so results are reported separately for Māori and Non-Māori (although derived from the same models).

Nonetheless, similar multivariable models were subsequently run for children identifying as Māori and Non-Māori, with ΔcBMI as a continuous variable as the main predictor of interest. Additional 2-way interactions were examined (ΔcBMI*child’s sex and ΔcBMI*child’s age), with interaction terms subsequently removed if not significant.

Data are reported as *β* coefficients and respective 95% confidence intervals (CI) for continuous associations. For stratified analyses, data are provided as least-squares means (i.e. adjusted means) and adjusted mean differences (aMD), with respective 95% CI. Statistical tests were two-sided with significance at *p* < 0.05; p-values for within-group differences from stratified analyses are referred to as “pWG” in the text to differentiate these from the primary *p* values for between-group differences. Data were analysed in SAS v9.4 (SAS Institute, Cary, North Carolina, USA), and figures drawn in GraphPad Prism v8.4.3 (GraphPad Software Inc., San Diego, CA, USA).

## Results

Two hundred thirty-nine children and adolescents were referred during the recruitment period; 231 had BMI SDS recorded at baseline, and 95% had a caregiver who agreed to have their weight recorded. Due to the presence of siblings, 203 individual adults (mostly female) had BMI recorded at baseline, 107 at 12 months, and 86 at 24 months (Supplementary Table 1).

At baseline, the caregiver’s BMI was positively correlated with their child’s BMI SDS (*r* = 0.25; *p* < 0.001). The multivariable model showed that a 1.0 kg/m^2^ increase in caregiver’s BMI was associated with a 0.016 SDS increase in their child’s BMI [*β* = 0.016 (0.006, 0.026); *p* = 0.003].

Overall, there were no changes in mean caregiver’s BMI at 12 months [0.04 kg/m^2^ (−0.47, 0.56); *p* = 0.88] or at 24 months [0.47 kg/m^2^ (−0.21, 1.14); *p* = 0.17]. At 12 and 24 months, interaction terms in stratified analyses showed the association between ΔcBMI group and the child’s ΔBMI SDS differed in children identifying as Māori and non-Māori.

**Fig. 1:**
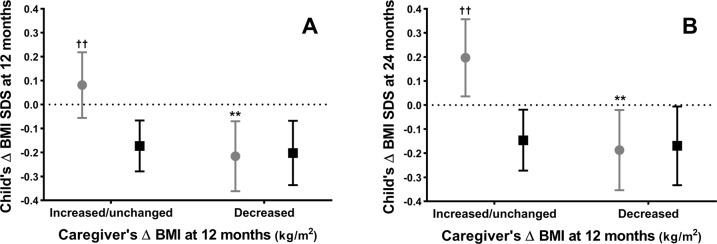
Changes (Δ) in body mass index standard deviation score (BMI SDS). Changes (Δ) in BMI SDS from baseline at 12 months (**A**) and 24 months (**B**) among children identifying as Māori (grey circles) and Non-Māori (black squares) whose caregiver’s BMI was reduced or increased/unchanged at 12 months. Data are the least-squares means (i.e. adjusted means) with error bards representing the respective 95% confidence intervals, which were derived from linear mixed models adjusting for caregiver ΔBMI group, ethnicity, and their interaction term, as well as sex, household deprivation, the child’s BMI SDS and age at baseline, with family ID included as a random factor. **p < 0.01 for the comparison between Māori children from the two caregiver ΔBMI groups at a given time-point; ^††^*p* < 0.01 for the comparison between Māori and Non-Māori children within a given caregiver Δ BMI group at a given time-point.

**Fig. 2:**
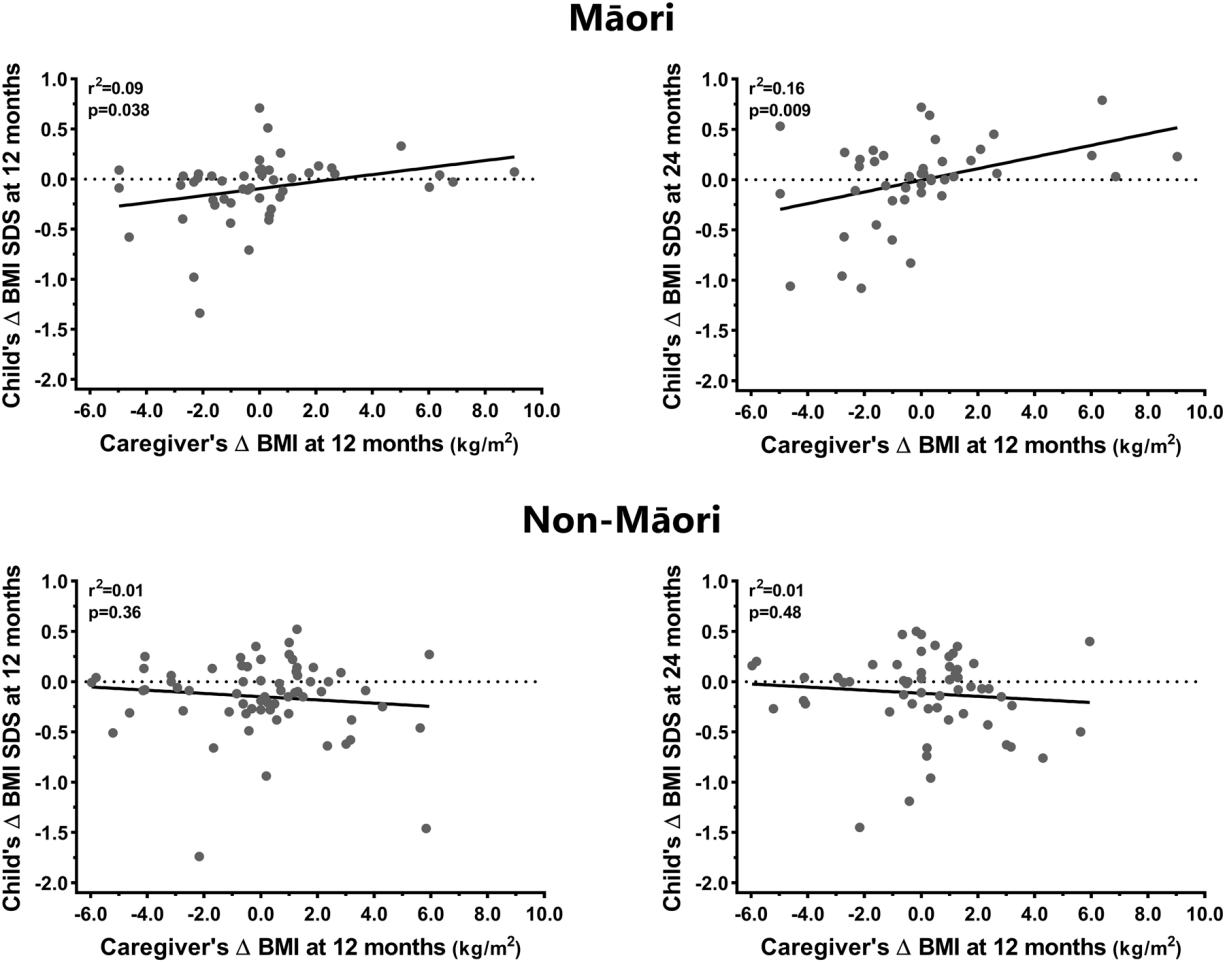
Linear associations between the changes (Δ) in body mass index (BMI) from baseline among caregivers of participants identifying as Māori and Non-Māori at 12 months and their children’s Δ BMI standard deviation score (SDS) at 12 and 24 months. Simple regression lines are shown with respective coefficients (*r*^2^) and *p* values. Horizontal dotted lines represent the children’s reference BMI SDS values at baseline.

Among Non-Māori children, stratified analyses yielded an average reduction in BMI SDS at both 12- and 24-month assessments (Supplementary Table 2; Supplementary Fig. 1), irrespective of ΔcBMI (Fig. 1). Consequently, for Non-Māori participants there were no observed associations between ΔcBMI and their child’s ΔBMI SDS, at 12 months [*β* = −0.003 (−0.039, 0.032); *p* = 0.09] or 24 months [*β* = 0.010 (−0.051, 0.032); *p* = 0.06] (Fig. 2).

In contrast, among children identifying as Māori at 12 months, BMI was 0.22 SDS lower than baseline (95% CI −0.36, −0.07; pWG = 0.002) in the group whose caregivers had reduced their BMI, whereas mean BMI SDS did not change [0.08 (95% CI −0.06, 0.22); pWG=0.24] among children of caregivers with increased or unchanged BMI [aMD −0.30 (95% CI −0.49, −0.10); *p* = 0.004) (Fig. 1; Supplementary Fig. 1). There was a similar pattern at 24 months; children identifying as Māori whose caregivers’ BMI had decreased at 12 months maintained a mean BMI reduction of −0.19 SDS (95% CI −0.35, −0.02; pWG = 0.029), while average BMI increased by 0.20 SDS (95% CI 0.04, 0.36; pWG = 0.017) among Māori children of caregivers whose BMI had not changed or increased [aMD −0.39 (95% CI −0.61, −0.16); *p* = 0.001] (Fig. 1; Supplementary Fig. 1).

Among children identifying as Māori, a reduction in caregiver’s BMI at 12 months was associated with reductions in their child’s BMI SDS at 12 months (*r* = 0.30; *p* = 0.038) and 24 months (*r* = 0.39; *p* = 0.009) (Fig. 2). At 12 months, a 1.0 kg/m^2^ reduction in caregiver’s BMI was associated with a 0.033 SDS decrease in their child’s BMI [β = 0.033 (0.000, 0.066); *p* = 0.049]. At 24 months however, there was an interaction between the child’s age at baseline and ΔcBMI at 12 months; the younger the child was at recruitment, the greater was the ΔBMI SDS magnitude at 24 months in association with a marked reduction or a marked increase in caregiver’s BMI at 12 months (Supplementary Fig. 2). Conversely, for children identifying as Māori aged approximately 12 years or older at baseline, ΔcBMI appeared to have limited impact on ΔBMI SDS at 24 months (Supplementary Fig. 2).

## Discussion

When examining the caregivers’ BMI of children enrolled in this family-centred programme for childhood obesity, there was no indirect reduction of BMI overall. However, associations were found between changes in caregivers’ BMI and changes in their children’s BMI SDS. A BMI SDS reduction was observed for non-Māori children at 12 and 24 months, while for children identifying as Māori, a BMI SDS reduction was noted if a decrease in caregiver’s BMI was achieved.

The association between caregiver’s BMI change at 12 months and the child’s change in BMI SDS at both 12 and 24 months emphasises the importance of a culturally relevant, family-centred approach for participants identifying as Māori when addressing weight issues and working to achieve healthy lifestyle changes. Focus groups with participants and families from Whānau Pakari have echoed our findings, stressing the importance of connectedness and family-focused programmes that support families to become self-determining in their process to achieve healthy lifestyle changes [13]. This aligns with a *Whānau Ora* approach, which is based on a Māori philosophy where *whānau* (family – immediate or wider) enablement is considered fundamental for the wellbeing of individuals within the family [14]. We do not have data to explain the different associations between caregivers’ changes in BMI and their children’s change in BMI SDS, and further research would be required to understand the underlying reasons for these findings for children identifying as Māori. Nonetheless, previous participants indicated a sense of difference with the Whānau Pakari programme compared with more conventional interactions with the healthcare system, which were often negative and delivered in a less culturally appropriate manner [15, 16]. Due to Whānau Pakari’s focus on compassionate, respectful care and emphasis on positive relationship-building, participants identifying as Māori reported that the programme was culturally appropriate, creating an environment where healthy lifestyle change was possible and considered achievable [15], which may, in part, explain why changes in BMI SDS were achieved when caregivers also reduced their BMI.

The finding that overall caregivers’ BMI did not decrease in the RCT was not surprising. There were no targeted efforts in the programme focusing specifically on caregiver’s weight, but instead aiming to achieve healthy lifestyle changes for the whole family. The changes in BMI of caregivers observed in previous studies may have been due to their more direct focus on caregiver’s weight in these programmes [8, 9]. The observed benefits from reductions in the caregivers’ BMI indicate that increased support targeting the caregivers’ weight status may lead to improvements for the children, particularly among those identifying as Māori.

The present study achieved a strong representation of those most affected by obesity, with a high representation of Māori (46%) and individuals with high levels of household deprivation. Compared to previous investigations [8, 9], this study undertook a longer duration of follow-up, giving insight into the long-term impact of the intervention. A noted limitation was the loss of accompanying adult data over time. Additionally, data collection for accompanying adults in the programme was entirely voluntary and might not be representative of the whole cohort.

In conclusion, a reduction in caregivers’ BMI was an important factor associated with improvements in BMI SDS among children identifying as Māori. This study confirms the importance of culturally relevant, family-based intervention programmes and seeing the individual within a broader context, particularly for those most affected by obesity. There is a clear need for further research on additional ways to support parents and caregivers to achieve healthy lifestyle changes in such programmes. Given the intergenerational nature of obesity, such strategies may contribute to clinically meaningful improvements in weight status across the entire family.

## Supplementary information

Supplementary File

## Data Availability

The study data cannot be made available in a public repository due to the strict conditions of the ethics approval, as no consent was obtained from study participants to make their confidential health data publicly available, even if anonymised. Nonetheless, the anonymised data on which this manuscript was based could be made available to other investigators upon bona fide request, and following all the necessary approvals (including ethics approval) of the detailed study proposal and statistical analyses plan. Any queries should be directed to YCA (y.anderson@auckland.ac.nz).
