# Supplementary material for: Associations between changes in caregiver’s and child’s weight status in a community-based obesity intervention programme
Source: Int J Obes (Lond). 2022 Apr 29;46(7):1406–9. doi: 10.1038/s41366-022-01121-3 (PMC9239902; doi:10.1038/s41366-022-01121-3)
Supplement: Supplementary file 1 — Supplementary File [file 41366_2022_1121_MOESM1_ESM.pdf]

SUPPLEMENTARY FILE

Associations between changes in caregiver's and child's weight status in a community-based obesity intervention programme

Thomas S Hadley[1], Tami L Cave[2], José G B Derraik[2,3], Paul L Hofman[2,4], Yvonne C Anderson[1,3,5,6,7]

[1] Department of Paediatrics, Taranaki District Health Board, New Plymouth, New Zealand

[2] Liggins Institute, University of Auckland, Auckland, New Zealand

[3] Department of Paediatrics: Child and Youth Health, Faculty of Medical and Health Sciences, University of Auckland, Auckland, New Zealand

[4] Starship Children's Health, Auckland District Health Board, Auckland, New Zealand

[5] enAble Institute, Faculty of Health Sciences, Curtin University, Bentley, WA, Australia

[6] Telethon Kids Institute, Perth Children's Hospital, Nedlands, WA, Australia

[7] Community Health, Child and Adolescent Health Service, Perth, WA, Australia

Corresponding author: Dr Yvonne Anderson, Department of Paediatrics: Child and Youth Health, Faculty of Medical and Health Sciences, University of Auckland, Private Bag 92019, Auckland 1142, New Zealand. Email: y.anderson@auckland.ac.nz

Supplementary Table 1

Characteristics of participants in the Whānau Pakari randomised controlled trial and their accompanying adults at the baseline, 12-month, and 24-month assessments.

			Baseline	12 months	24 months
**Children**	**n**		220	116	100
	**Female**		118 (53.6%)	60 (52%)	52 (52%)
	**Age (years)**		10.8 [8.3, 13.2]	11.5 [8.9, 13.6]	12.4 [9.8, 14.7]
	**Ethnicity [a]**	**Māori**	101 (46%)	48 (41%)	44 (43%)
		**Pacific**	5 (2%)	2 (2%)	–
		**European**	105 (48%)	62 (53%)	49 (49%)
		**Asian**	9 (4%)	4 (3%)	8 (8%)
	**Deprivation quintiles [b]**	**1 (least deprived)**	22 (10%)	14 (12%)	13 (13%)
		**2**	33 (15%)	18 (16%)	19 (19%)
		**3**	44 (20%)	30 (26%)	25 (25%)
		**4**	58 (26%)	31 (27%)	24 (24%)
		**5 (most deprived)**	63 (29%)	23 (20%)	19 (19%)
	**BMI SDS**		3.12 [2.68, 3.43]	2.95 [2.39, 3.34]	2.97 [2.44, 3.40]
**Adults**	**n**		203	107	86
	**Relationship to child**	**Mother**	166 (82%)	95 (89%)	75 (87%)
		**Father**	23 (11%)	7 (7%)	7 (8%)
		**Other caregiver**	14 (7%)	5 (5%)	4 (5%)
	**BMI (kg/m^2)**		32.5 [27.9, 38.1]	32.3 [28.0, 38.0]	32.7 [28.1, 37.9]

Data are n (%) or median [quartile 1, quartile 3], as appropriate.
[a] Prioritised ethnicity.
[b] Levels of household deprivation stratified into quintiles based on the New Zealand Deprivation Index 2006.
BMI, body mass index; BMI SDS, body mass index standard deviation score.

Supplementary Table 2

Changes (Δ) in body mass index standard deviation score (BMI SDS) from baseline at 12 and 24 months among Non-Māori participants whose caregivers' BMI were reduced or increased/unchanged at 12 months.

		Accompanying adult's Δ BMI from baseline		
		**Increased/Unchanged**	**Reduced**	**aMD**
**12 months**	**n**	41	27	
	**Δ BMI SDS**	-0.17 (-0.28, -0.07) ††	-0.20 (-0.34, -0.07) ††	0.03 (-0.14, 0.20)
**24 months**	**n**	35	21	
	**Δ BMI SDS**	-0.15 (-0.27, -0.02) †	-0.17 (-0.33, -0.01) †	0.02 (-0.18, 0.23)

aMD –adjusted mean differences for Increased/Unchanged vs Reduced BMI.
Data are the least squares means (adjusted means) and the respective 95% confidence intervals derived from linear mixed models, which were adjusted for the participant's BMI SDS and age at baseline, ethnicity (Māori / Non-Māori), gender, household deprivation (NZDep2006), as well as family ID as a random factor.
†p<0.05 and ††p<0.01 for a statistically significant within-group change in the participants' BMI SDS from baseline.

Supplementary Figure 1

Changes (Δ) in body mass index standard deviation score (BMI SDS) from baseline at 12 and 24 months among children identifying as Māori and Non-Māori, whose caregivers' BMI were reduced or increased/unchanged at 12 months.

Horizontal lines represent the median and interquartile range, while the horizontal dotted lines represent the children's BMI SDS values at baseline. P-values were derived from linear mixed models, adjusted for caregiver Δ BMI group, ethnicity, and their interaction term, sex, household deprivation, and the child's BMI SDS and age at baseline, with family ID also included as a random factor.

Māori

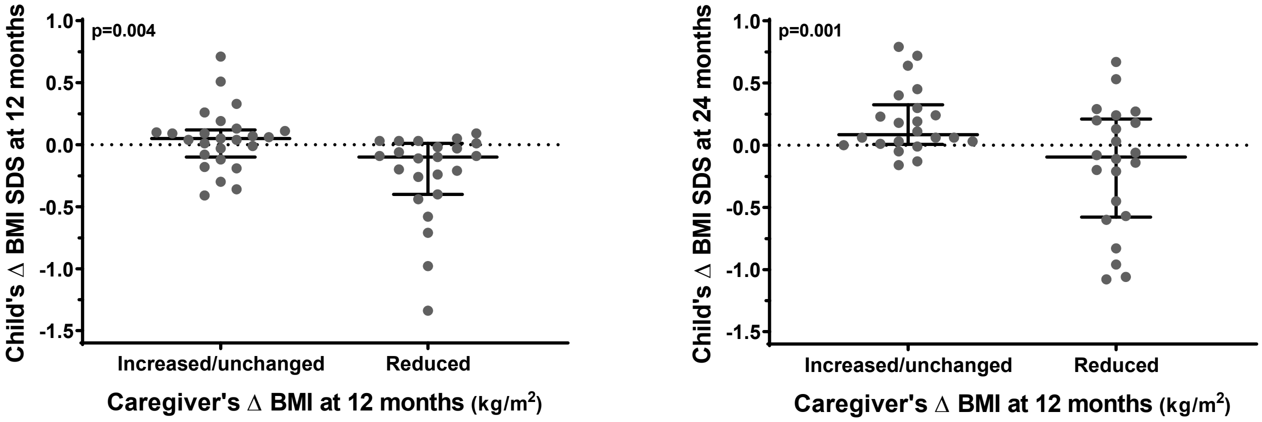

Non-Māori

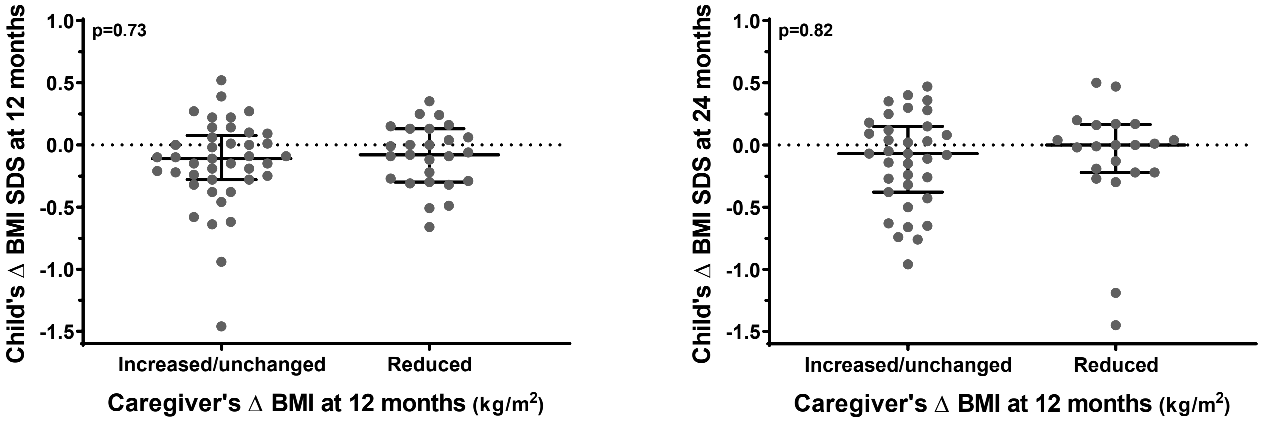

Hadley TS, Cave TL, Derraik JGB, Hofman PL, Anderson YC. Associations between changes in caregiver's and child's weight status in a community-based obesity intervention programme. *International Journal of Obesity*.

Supplementary Figure 2

Contour plot displaying the interaction between Māori caregiver's change (Δ) in body mass index (BMI) at 12 months and their child's age at baseline, and how this affected the child's change in BMI standard deviation score (BMI SDS) at 24 months (whose bands are represented by the contour lines).

Data were analysed using a linear mixed model, adjusted for caregiver Δ BMI group, child's age at baseline, their interaction term, sex, and household deprivation, as well as family ID as a random factor to account for the non-independence of siblings.

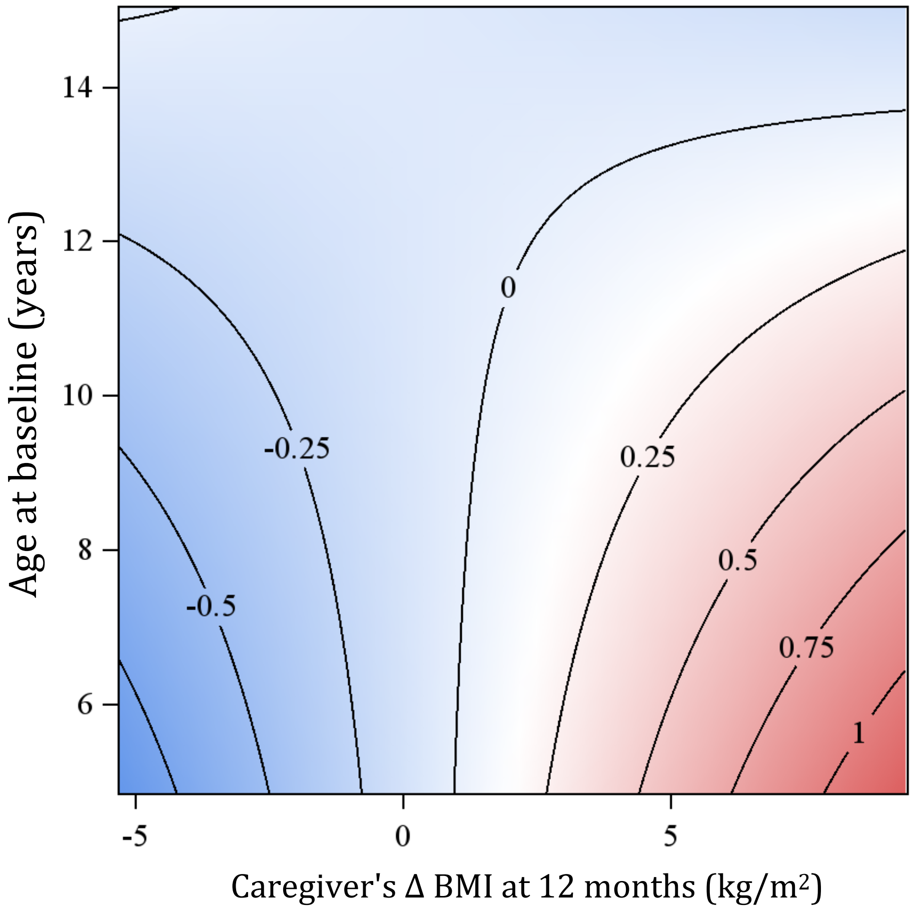

Hadley TS, Cave TL, Derraik JGB, Hofman PL, Anderson YC. Associations between changes in caregiver's and child's weight status in a community-based obesity intervention programme. *International Journal of Obesity*.
